# Educational inequalities, urbanicity and levels of non-communicable diseases risk factors: evaluating trends in Argentina (2005–2013)

**DOI:** 10.1186/s12889-021-11617-8

**Published:** 2021-08-20

**Authors:** Santiago Rodríguez López, Usama Bilal, Ana F. Ortigoza, Ana V. Diez-Roux

**Affiliations:** 1 Centro de Investigaciones y Estudios sobre Cultura y Sociedad, Consejo Nacional de Investigaciones Científicas y Técnicas (CIECS, CONICET y UNC), Córdoba, Argentina; 2 Cátedra de Antropología, Facultad de Ciencias Exactas, Físicas y Naturales, Universidad Nacional de Córdoba (FCEFyN – UNC), Córdoba, Argentina; 3 Urban Health Collaborative, Dornsife School of Public Health, Drexel University, Philadelphia, USA; 4 Department of Epidemiology and Biostatistics, Dornsife School of Public Health, Drexel University, Philadelphia, USA

**Keywords:** Inequities, Urbanization, Health, Diabetes, Hypertension, Obesity, Tobacco, Adults

## Abstract

**Background:**

We investigated a) whether urbanicity is associated with individual-level non-communicable diseases (NCD) risk factors and whether urbanicity modifies trends over time in risk factors; and (b) whether educational inequalities in NCD risk factors change over time or are modified by province urbanicity.

**Methods:**

We used data from three large national surveys on NCD risk factors (Encuesta Nacional de Factores de Riesgo; ENFR_2005–2009-2013_) conducted in urban areas of Argentina (*n* = 108,489). We used gender-stratified logistic random-intercept models (individuals nested within provinces) to determine adjusted associations of self-reported individual NCD risk factors (hypertension, diabetes, obesity, and current smoking) with education and urbanicity.

**Results:**

In both men and women, the prevalence of obesity and diabetes increased over time but smoking decreased. Hypertension prevalence increased over time in men. Higher urbanicity was associated with higher odds of smoking and lower odds of hypertension in women but was not associated with NCD risk factors in men. Obesity increased more over time in more compared to less urbanized provinces (in men) while smoking decreased more over time in less urbanized provinces. All risk factors had a higher prevalence in persons with lower education (stronger in women than in men), except for diabetes in men and smoking in women. Educational inequalities in obesity (in men) and hypertension (in men and women) became stronger over time, while an initial inverse social gradient in smoking for women reverted and became similar to other risk factors over time. In general, the inverse associations of education with the risk factors became stronger with increasing levels of province urbanicity.

**Conclusion:**

Increasing prevalence of diabetes and obesity over time and growing inequities by education highlight the need for policies aimed at reducing NCD risk factors among lower socioeconomic populations in urban environments in Argentina.

## Background

Most research on risk factors for non-communicable diseases (NCDs) has focused on individual-level risk factors, but over the past few years there has been an increased focus on macro-level contextual factors [1]. Macro-level and contextual factors affect individual health by exposing people to different levels of social and economic opportunities and constraints [2–4]. These exposures, in turn, may shape individuals’ behavioral patterns, stress levels and coping resources, which are directly relevant to their health outcomes [5].

Urbanization, a process by which populations migrate from rural to urban areas has consequences for health [6]. Whether these consequences are positive or negative depends on a number of factors, including specific contexts, times, and health outcomes [7]. Although greater urbanization is usually related to a number of benefits, such as higher income, greater access to services, and lower poverty [8], at least, in some contexts, increased urbanization is associated with adverse health outcomes [9]. For example, research shows that in many developing countries, those living in urban areas have higher levels of NCDs than do their rural counterparts [10, 11]. Additionally, rapid and recent urbanization has been associated with unhealthy changes in diet, lower physical activity, more smoking and alcohol consumption, and higher inflammation [10, 12–14].

Only a few studies have examined macro and contextual determinants of NCD risk factors or inequalities in NCD risk factors in countries of Latin America, one of the most urbanized regions in the world [15]. At around 90% urbanicity, Argentina is one of the most urbanized countries worldwide [16]. Previous research in Argentina has investigated differences in the prevalence of NCD risk factors [17, 18] and variations in NCD risk factors by socioeconomic position (SEP) [19, 20].

The extent to which socioeconomic inequalities in NCD risk factors vary across contexts of urbanization is less known. Fleischer et al. used cross-sectional data from 2005 to investigate whether social inequalities in NCD risk factors were modified by province level urbanicity in Argentina. The authors reported stronger inverse gradients between SEP and NCD risk factors in more urban than in less urban contexts [21]. Christine et al. used data from 2005 and 2009 to explore whether changes over time in population mean body mass index (BMI) were modified by province-level economic development, which is often closely linked to urbanization. They found slightly greater mean annual increase in BMI occurring in provinces with greater economic growth [22]. However, to our knowledge, no studies have investigated how urbanicity relates to changes in multiple NCD risk factors over time or to changes in educational inequalities in NCD risk factors over time.

We used three Argentinean large population surveys linked to a province level indicator to investigate (a) whether province urbanicity is associated with individual-level risk factors for NCDs and whether urbanicity modifies trends over time in risk factors; and (b) whether inequalities by individual-level education in NCD risk factors change over time or are modified by province urbanicity. We hypothesized that: (1) compared to individuals from provinces with lower urbanization, individuals from provinces with higher urbanization have greater NCD risk; (2) provinces with higher urbanization have experienced less favorable trends over time in NCD risk; (3) inequalities by individual-level education in NCD risk factors have decreased over time; (4) provinces with higher urbanization have greater inequalities by individual-level education in NCD risk factors.

## Methods

### Sample

Data used in this study included three large repeat cross-sectional surveys conducted as part of the Argentine National Survey of Risk Factors (‘Encuesta Nacional de Factores de Riesgo’, ENFR), carried out in urban areas of Argentina in 2005, 2009, and 2013. The ENFR is part of the Non-Communicable Diseases Surveillance System and the Integrated System of Household Surveys of Argentina. ENFRs are carried out every 4 years in agreement with the National Ministry of Health, the National Institute of Statistics and Censuses, and Provincial Directorates of Statistics. They include information on the housing conditions and socioeconomic and demographic characteristics of the head of the household, together with individual information on self-reported health, NCD and cardiovascular RF prevalence [18]. Further methodological details of the ENFRs can be found elsewhere [23], but a summary follows. The ENFRs are provincial and nationally representative samples of urban adults, specifically of the non-institutionalized population aged 18 years or more living in localities with 5000 or more residents [19]. Sample sizes (and response rates) of the ENFRs were 41,392 (86.7%), 34,732 (79.8%), and 32,365 (70.7%) for ENFR_2005_, ENFR_2009_, and ENFR_2013_, respectively. Each wave of the ENFR samples individuals independently from previous waves, using a cross-sectional probabilistic multistage sample design (visit https://www.indec.gob.ar/bases-de-datos.asp for more details). The overall pooled sample for this analysis included 108,489 individuals (age range: 18–98 years).

### Area-level variable

We investigated urbanicity, a time-varying province characteristic, as the contextual factor. In Argentina, over 90% of the population lives in urban areas, but there is wide heterogeneity within the country. Urban localities are defined by the National Institute of Statistics and Censuses as those of more than 2000 inhabitants [24], while those with less than 2000 inhabitants are defined as “grouped rural population” [25]. We used the percentage of households living in urban areas (areas of 2000 habitants or more) from the 2001 [24] and 2010 [16] Argentinean Census, as a proxy of urbanization [21]. We linked values from the 2001 and 2010 census to the ENFR_2005_ and ENFR_2013_, respectively, and performed a linear interpolation to estimate a value for 2005–2006, linked to the ENFR_2009_. Values were assigned to each respondent based on their province of residence and the year of the survey. Urbanicity was centered by the overall mean and scaled by the overall standard deviation.

### Individual-level variables

Individual-level variables included gender, age, and education as a proxy for individual SEP. Level of education was defined as 1. no formal education, 2. primary incomplete, 3. primary complete, 4. secondary incomplete, 5. secondary complete, 6. tertiary/university incomplete, 7. tertiary/university complete.

Educational inequalities were quantified using the relative index of inequality (RII) [26]. The RII is a regression-based measure that resembles a relative risk in that it compares the health of the extremes of the social distribution, but it is estimated using data from all social categories [27, 28]. To calculate the RII, first the educational groups were transformed into cumulative rank probabilities (ridit scores) ranging from 0 (highest level of education) to 1 (lowest level of education). A modified ridit score was assigned to the population in each education category, based on the mid-point of the range in the cumulative distribution of the individuals in the given categories [29]. Weighted ridit scores for individual educational level were generated for each survey separately, via the Stata *wridit* function [30]. Finally, the coefficient obtained in regression analyses (when the link function is logit) expressed the RII, which can be interpreted as the rate ratio between the least and the most educated people (i.e. an RII > 1 implies an inverse relationship between outcomes and education; RII < 1 implies a positive relationship between these variables).

### Outcomes

The four key outcomes of this study were self-reported dichotomous hypertension, obesity, diabetes, and current smoking for each individual in each survey. Hypertension and diabetes were defined as having been told by a health professional that one had high blood pressure or diabetes/high blood sugar. Obesity was defined based on a BMI above or equal to 30 kg/m^2^, computed based on self-reported height and weight. Current smoking was determined based on self-reported tobacco consumption.

### Statistical analyses

Descriptive statistics accounted for the complex sample design of the survey by including weights in calculation of means/percentages. We performed all subsequent unweighted, since our intent was to estimate associations between a contextual province factor and NCD risk factors in the study sample, rather than to provide estimates of the prevalence of risk factors in the general population [31]. Variables relevant to weights (like age and gender) were included as adjustment factors.

We fitted two-level logistic random-intercept models of individuals nested within provinces. Since respondent samples differed by province over time and in order to account for time trends, calendar time was included as a province-level characteristic. Analyses were conducted using the *melogit* command in Stata 14 [32]. We stratified all analyses by gender. We fitted final models informed by the proposed theoretical conceptualization, as follows:

$$ logit\ of\ {P}_{ijt}={\gamma}_{00}+{\gamma}_{01}{U}_{jt}+{\gamma}_{02}{S}_{jt}+{\gamma}_{03}{U}_{jt}{S}_{jt}+{\gamma}_{10}{A}_{ijt}+{\gamma}_{20}{E}_{ijt}+{\gamma}_{21}{S}_{jt}{E}_{ijt}+{\gamma}_{22}{U}_j{E}_{ijt}+{\vartheta}_j $$

Where *P*_*ijt*_ is the probability of the outcome for person *i* at province *j,* survey time *t*; *γ*_01_ is the main effect of province urbanicity (hypothesis 1); *γ*_02_ is the main effect of survey calendar time (included as two dummies with the first year being the reference); *γ*_03_ is the interaction between province urbanicity and survey (hypothesis 2); *γ*_10_ is the main effect of age; *γ*_20_ is the main effect for education (i.e. the exponentiated coefficient is the RII); *γ*_21_ is the interaction between survey and education (shows potential trends in educational inequalities; hypothesis 3); *γ*_22_ is the interaction between province urbanicity and education (hypothesis 4); *ϑ*_*j*_ is the random effect for province.

We explored four models of increasing complexity for each outcome. First, an empty model with no explanatory variables in the fixed part and a random variance component for provinces. The random part suggested whether variations existed between provinces, and if this variation remained statistically significant when controlling for individual and province-level predictors in subsequent models. Model 1 added individual variables of age and education (ridit scores) to the empty model. Model 2 added province urbanicity as well as survey time to Model 1. This model formally tested whether contextual urbanicity was associated with individual risk of NCD risk factors after adjusting for age, education and time (hypothesis 1). This model also showed inequalities by individual-level education for each outcome (RII), after controlling for age, province urbanicity, and time. Finally, model 3 included interaction terms to test (a) whether provinces with higher urbanicity have experienced less favorable trends over time in NCD risk factors (coefficient *γ*_03_ above) (hypothesis 2); (b) whether inequalities by individual-level education (RII) changed over time (coefficient *γ*_21_ above) (hypothesis 3); (c) whether inequalities by individual-level education (RII) were modified by province-level urbanicity (coefficient *γ*_22_ above) (hypothesis 4). Each interaction term was included in separate models (i.e. interactions were added one by one). Interactions between urbanicity and other factors (calendar time and individual-level education) were depicted graphically by showing associations for low (10th percentile) and high (90th percentile) levels of province urbanization.

## Results

Table 1 shows the distribution across provinces and general trends over time in the studied variables. Hypertension and diabetes increased slightly over time. The prevalence of obesity increased over time and the prevalence of smoking decreased over time. Education showed slight improvements over time, with fewer individuals in lower categories of education, and more in the higher categories. Additionally, the overall percentage of households living in urban areas showed an increment over time (Table 1).

**Table 1:** Characteristics of the study population by survey year: National Survey of Risk Factors (ENFR) 2005, 2009, and 2013, Argentina

	ENFR_**2005**_	ENFR_**2009**_	ENFR_**2013**_
	(***n*** = 41,392)	(***n*** = 34,732)	(***n*** = 32,365)
Sample size per province, median	1670	1271	1051
***Individual-level characteristics***			
Female, %	52.5	53.3	52.6
Age, mean (SD)	43.3 (17.9)	43.6 (18.0)	44.3 (17.9)
Education, %			
No formal education	1.8	1.6	1.3
Primary incomplete	11.1	9.2	8.6
Primary complete	26.2	22.7	21.6
Secondary incomplete	16.8	17.3	16.7
Secondary complete	20.1	22.4	24.5
Tertiary/University incomplete	11.9	12.2	11.7
Tertiary/University complete	12.1	14.6	15.6
Outcomes, %			
Hypertension	31.2	34.8	34.3
Diabetes	8.5	9.6	9.8
Obesity	15.8	18.5	21.5
Current smoking	29.7	27.1	25.1
***Province-level characteristic***			
Households living in urban areas, mean (SD)	84.8 (7.8)	87.4 (7.3)	88.1 (6.9)

Twenty-four geographical/administrative units (twenty-three provinces and the City of Buenos Aires) represented in each survey

Table 2 shows associations of NCD risk factors with province-level urbanicity, individual education and survey years (Model 2). Province-level urbanicity was not associated with any NCD risk factors in men. However, 1SD higher in province urbanicity (7.5%) was associated with lower odds of hypertension (OR 0.92) and higher odds of current smoking (OR 1.13) among women (Table 2).

**Table 2:** Odds ratios of hypertension, diabetes, obesity, and current smoking by education, province urbanicity, and year of survey (Model 2)

	Hypertension	Diabetes	Obesity	Current smoking
	**OR (95% CI)**			
***Men***	(*n* = 42,122)	(*n* = 46,804)	(*n* = 44,817)	(*n* = 47,083)
Education, RII	**1.26 (1.16, 1.37)**	0.94 (0.83, 1.06)	**1.39 (1.27, 1.52)**	**1.99 (1.85, 2.15)**
Urbanicity, SD	1.01 (0.96, 1.06)	0.99 (0.92, 1.07)	1.02 (0.95, 1.08)	1.03 (0.98, 1.07)
Survey (ref. 2005)	1.00	1.00	1.00	1.00
2009	**1.19 (1.13, 1.26)**	**1.17 (1.08, 1.27)**	**1.18 (1.11, 1.25)**	**0.80 (0.77, 0.84)**
2013	**1.16 (1.09, 1.23)**	**1.14 (1.05, 1.24)**	**1.45 (1.36, 1.54)**	**0.70 (0.66, 0.73)**
*Random effects*				
Intercept variance (Std. error)	0.012 (0.005)	0.026 (0.010)	0.022 (0.008)	0.009 (0.003)
***Women***	(*n* = 58,177)	(*n* = 61,075)	(*n* = 55,733)	(*n* = 61,247)
Education, RII	**2.41 (2.26, 2.57)**	**1.97 (1.79, 2.17)**	**2.79 (2.57, 3.03)**	1.06 (0.99, 1.14)
Urbanicity, SD	**0.92 (0.88, 0.97)**	0.97 (0.90, 1.04)	0.99 (0.92, 1.07)	**1.13 (1.03, 1.23)**
Survey (ref. 2005)	1.00	1.00	1.00	1.00
2009	1.02 (0.98, 1.07)	**1.20 (1.12, 1.28)**	**1.17 (1.11, 1.24)**	**0.78 (0.75, 0.82)**
2013	1.03 (0.98, 1.08)	**1.21 (1.13, 1.29)**	**1.41 (1.33, 1.49)**	**0.70 (0.67, 0.74)**
*Random effects*				
Intercept variance (Std. error)	0.014 (0.005)	0.029 (0.010)	0.031 (0.010)	0.053 (0.017)

Age-adjusted analyses; RII, relative index of inequality (RII > 1 indicates higher prevalence with lower levels of education, RII < 1 indicates lower prevalence with lower levels of education); Urbanicity, percentage of households living in urban areas (per SD = 7.5). Model for each outcome include all main effects of the variables simultaneously with no interactions (Model 2)

Changes in the prevalence of obesity, diabetes and smoking over time showed a similar pattern in men and women. While the prevalence of obesity and diabetes consistently increased over time, smoking consistently decreased (Table 2). Additionally, the prevalence of hypertension increased over time in men (especially between ENFR_2005_ and ENFR_2009_) but did not change substantially in women. The random components of the models suggested that variations across provinces were rather small but statistically significant (empty and model 1, not shown), and that these variations remained statistically significant after controlling for province urbanicity (Model 2, Table 2).

The educational patterning of hypertension, diabetes and obesity was substantially stronger in women than in men. Lower education was strongly associated with higher odds of hypertension (RII 2.41), diabetes (RII 1.97), and obesity (RII 2.79) in women. Lower education (age-adjusted RII) was also associated with higher odds of hypertension (RII 1.26) and obesity (RII 1.39) in men but diabetes was not patterned by education in men. Lower education was associated with more smoking in men (RII 1.99) but no educational inequalities in smoking were observed in women (Table 2).

**Fig. 1:**
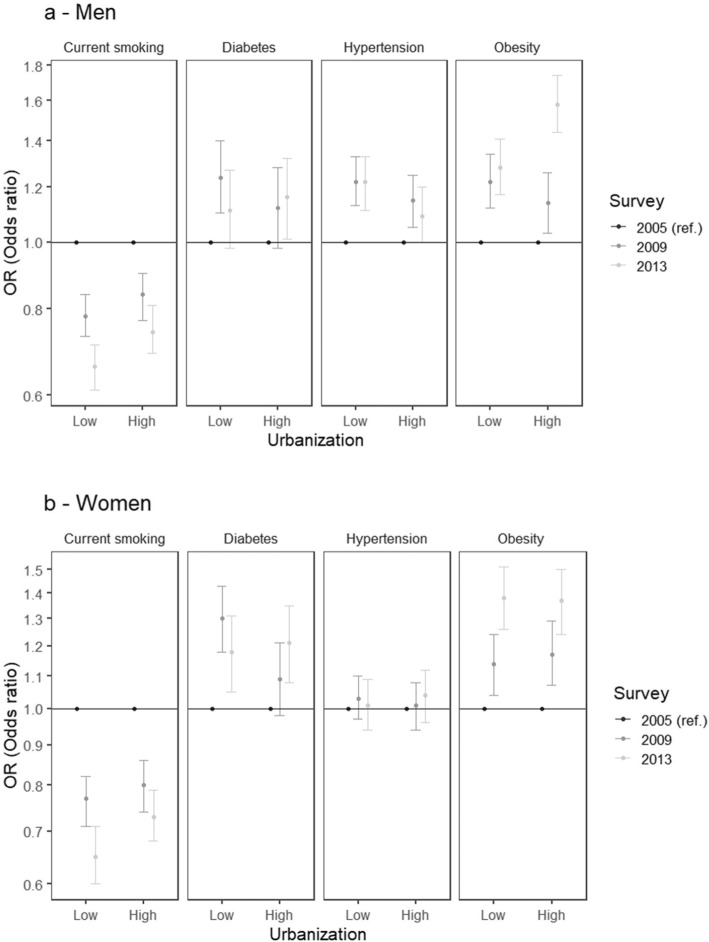
**a** and **b**. Adjusted odds ratios of hypertension, diabetes, obesity, and current smoking associated with survey year (with year 2005 as the reference) stratified by low (10th percentile) and high (90th percentile) province urbanicity in men (**a**) and women (**b**). Period 2005–2013. Model for each outcome includes all main effects and interaction for urbanicity and survey. Analyses are adjusted for age and education. Survey year 2005 is the reference category. Low and high province urbanicity correspond to 10th and 90th percentile, respectively. Significant p values for interactions include: Men, obesity (*p* = 0.001); Women, diabetes (*p* = 0.028)

Figure 1a and b show adjusted odds ratios of NCD risk factors associated with survey year (2005 as reference) for low (10th percentile) and high (90th percentile) province urbanicity in men and women. In men the increases over time in obesity tended to be larger in more compared to less urbanized provinces. Declines over time in smoking tended to be larger in less compared to more urbanized provinces (although tests for interaction were not statistically significant). No consistent differences in prevalence over time by level of urbanization were observed for hypertension or diabetes.

**Fig. 2:**
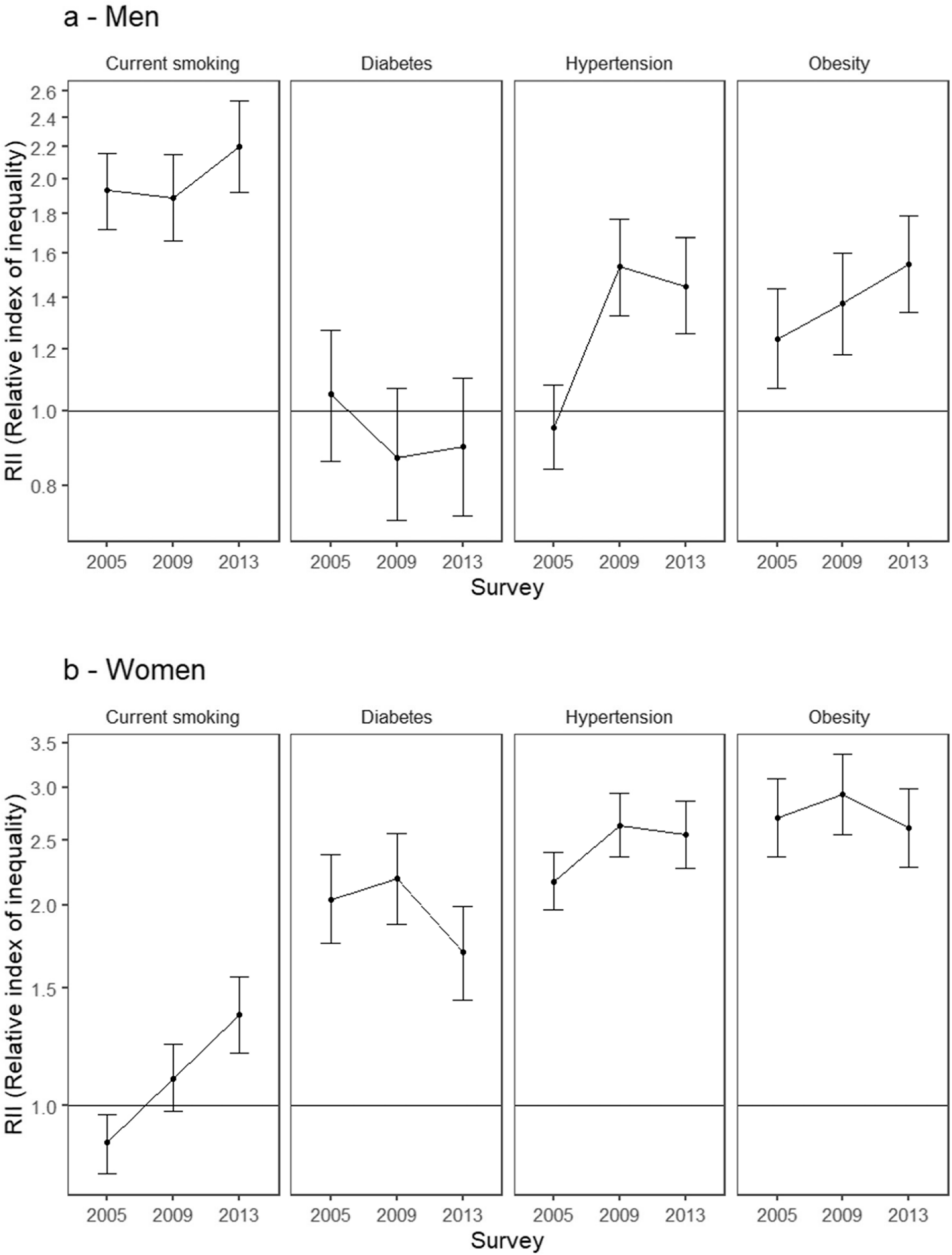
**a b**. Adjusted educational inequalities in hypertension, diabetes, obesity, and current smoking by survey year for (**a**) men and (**b**) women. Period 2005–2013. Model for each outcome includes all main effects and interaction for education and survey. Analyses are adjusted for age and urbanicity. RII > 1 indicates higher prevalence with lower levels of education, RII < 1 indicates lower prevalence with lower levels of education. Significant *p* values for interactions include: Men, hypertension (*p* < 0.001); Women, hypertension (*p* = 0.018) and current smoking (*p* < 0.001)

Figure 2a and b show adjusted educational inequalities in NCD risk factors by survey year. In men, educational inequalities in hypertension and obesity appeared to be larger in later survey years (although the p for interaction was statistically significant only for hypertension). In women (Fig. 2b), the association of low education with higher odds of hypertension became larger over time (global p for interaction < 0.05). In addition, the association of education with smoking changed direction over time: lower odds of smoking were observed in the lower education groups in 2005 but lower education groups had higher odds of smoking in 2013 (global p for interaction < 0.001).

**Fig. 3:**
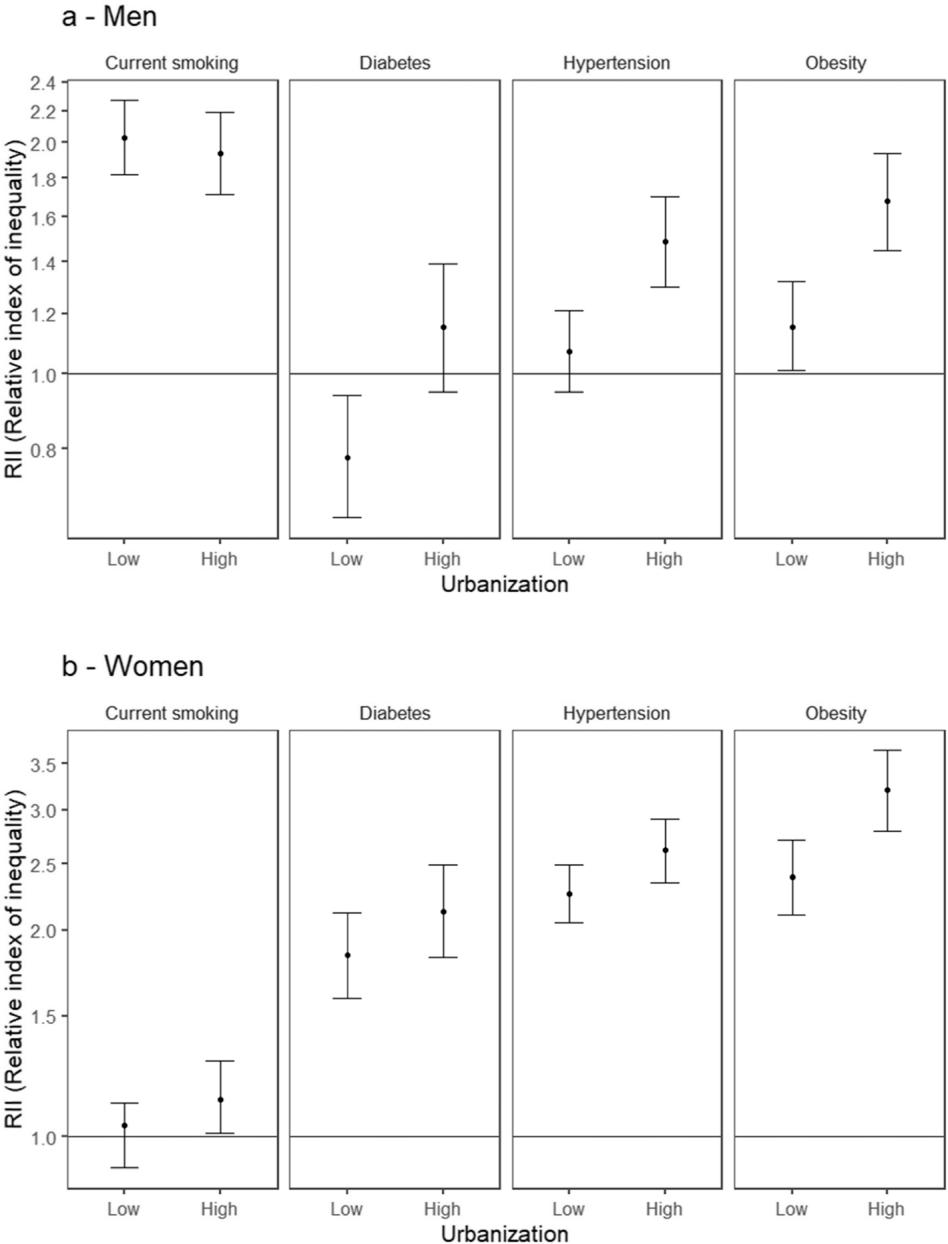
**a** and **b**. Adjusted educational inequalities in hypertension, diabetes, obesity, and current smoking for low and high province urbanicity for men (**a**) and women (**b**). Model for each outcome includes all main effects and interaction for education and urbanicity. Analyses are adjusted for age and survey year. RII > 1 indicates higher prevalence with lower levels of education, RII < 1 indicates lower prevalence with lower levels of education. Low and high province urbanicity correspond to 10th and 90th percentile, respectively. Significant p values for interactions include: Men, hypertension (*p* = 0.001), diabetes (*p* = 0.007) and obesity (*p* = 0.001); Women, obesity (*p* = 0.004)

Figure 3a and b show educational inequalities at low (10th percentile) and high (90th percentile) levels of province urbanicity. Among men (Fig. 3a), the association of lower education with higher odds of smoking was not modified by province urbanicity. However, for the other three risk factors (diabetes, hypertension, and obesity) the associations of lower education with higher odds of the risk factor became stronger (or only emerged) at higher levels of urbanicity (global p for interactions < 0.01 for diabetes and hypertension, and *p* < 0.001 for obesity). Among women (Fig. 3b), educational inequalities in all NCD risk factors were somewhat larger in provinces with high urbanicity, although the interaction term was only statistically significant for obesity (global p for interaction < 0.01). Notably, an association of low education with higher odds of smoking was present in provinces with higher urbanicity.

## Discussion

This study investigated trends and levels of individual-level educational inequalities in NCD risk factors for Argentinian adults, from 2005 to 2013, exploring how province-level urbanicity modified these inequalities and trends. We highlight five key findings. First, higher urbanicity was associated with higher odds of current smoking and with lower odds of hypertension in women, but was not associated with NCD risk factors in men. In both men and women, the prevalence of obesity and diabetes increased over time, but smoking decreased over time. Hypertension prevalence increased over time in men but did not change in women. Second, there was some evidence that obesity increased more over time in more compared to less urbanized provinces (in men) while smoking decreased more over time in less urbanized provinces. Third, we found a social gradient of higher prevalence of risk factors in people of lower education (stronger in women than in men), except for smoking in women and diabetes in men. Fourth, educational inequalities in obesity (in men) and hypertension (in men and women) became stronger over time, while the initial inverse social gradient in smoking for women reverted and became similar to other risk factors over time. Fifth, in general, the inverse associations of education with the risks factors became stronger as province urbanicity increased.

We hypothesized that individuals from provinces with higher urbanization would have greater NCD risk, compared to those living in less urbanized provinces. With the exception of smoking in women, we found no evidence that greater province urbanicity was associated with greater NCD risk factors in Argentina. The higher smoking prevalence in urban areas in women is consistent with other work reporting higher smoking prevalence in urban compared to non-urban areas in Europe [33] and may reflect differences in social norms and in access to tobacco products. Contrary to our hypothesis, higher urbanicity was associated with lower odds of hypertension among women.

Prior research has suggested that greater urbanization may be one of the main drivers of the rising burden of NCDs and cardiovascular risk factors [9, 34, 35]. It has been suggested that the links between urban living and NCD risk could be related to the greater access to unhealthy food and low physical activity [36, 37]. A systematic review of studies in low- and middle-income countries found that most but not all NCD risk factors (e.g. hypertension) were higher in recent rural-to-urban migrants than in rural groups, but lower than in urban groups [38]. Another study in Latin America [39] reported that the prevalence of obesity remained consistently higher among urban compared with rural individuals in most countries including Argentina, although obesity among rural populations is increasing at a faster pace than that among urban populations [40].

Argentina is a middle-high income country [41] so links between urbanization and NCD risk may be different from those observed in lower income countries at different stages of the epidemiologic transition [42]. Several other factors may have impacted our estimates of the associations of urbanicity with risk factors. Levels of urbanicity were generally high across provinces, so the range across which we could investigate this association was limited. We were not comparing rural to urban areas but rather provinces with already high levels of urbanization. In addition, we did not investigate differences in individual level exposures to urban and rural environments because our urbanicity measure is at the province level and the sample was drawn from urban areas. Thus, our inferences are limited to the contextual effect of living in more or less urban provinces, within a relatively high level of urbanization.

Like other work [18, 19, 22, 39, 43–45] we documented increases over time in the prevalence of diabetes and obesity in Argentina. Hypertension also increased in men. Although it has been suggested that increases in diabetes rates in the period 2005–2009 could be due to better health care access [45], the simultaneous increase in obesity suggests that changes in weight could be driving these increases in obesity related risk factors. In contrast, smoking decreased over time in both men and women, a positive development that may be linked to the adoption of smoke-free policies and the implementation of tobacco-protective best practices in Argentina [20, 46]. Decreasing trends in smoking occurred in almost all Latin American countries between 2005 and 2015 [47].

Furthermore, we expected that higher province urbanicity would be associated with less favorable trends over time in NCD risk (hypothesis 2). This was partially confirmed for obesity in men, and for current smoking in men and women. Likewise, Christine et al. [22] described slightly greater increases in BMI occurring in provinces of Argentina with greater economic growth, another development indicator. Our study complemented Christine et al. [22] by analyzing trends in other NCD risk factors (like diabetes, hypertension, and current smoking), and by adding ENFR_2013_. We found that the declines over time in smoking were larger in less urbanized provinces. However, U.S. data showed that smoking prevalence was declining at a slower rate in rural than urban settings [48], which might be attributable to policy-level tobacco control and regulatory factors that disproportionately benefited urban areas [49].

Like other studies, we documented strong inequities in risks factors by individual-level education in both men and women, although these associations were stronger in women than in men (with the single exception of smoking). Other work has also reported socioeconomic gradients in obesity, diabetes and low physical activity [19] and smoking [18, 20] in Argentina. Notably we found striking gender-differences in the social patterning of smoking: in women, higher education was associated with more smoking whereas the opposite association was observed in men. This is consistent with prior work in Argentina in 2005 [50] and in several European countries [51–53]. We found no evidence of diabetes inequalities in men. Since today’s obesity inequalities likely lead to tomorrow’s diabetes inequalities, the absence of diabetes inequalities in men may reflect a recent transition in the social patterning of obesity in men that could lead to inequalities in diabetes in the future.

We hypothesized that inequalities by individual-level education in NCD risk factors would decrease over time. However, we found the opposite: educational inequalities in obesity (in men) and hypertension (in men and women) increased over time. In addition, the educational patterning of smoking in women changed over time from a positive association to an inverse association. The educational patterning of smoking in women by which smoking is concentrated in women with lower education emerged in the 80’s in the U.S. [54] and in the 90’s in countries of Western Europe such as Spain [55] and Italy [51]. It has been posited that economic development and social–cultural processes related to gender empowerment have affected smoking habits in different ways for more and less educated women [56].

Consistent with our hypothesis, we found that the associations of lower education with higher risk factor prevalences –with the exception of current smoking among men- were larger for provinces with higher urbanization. Other studies have found that the socioeconomic patterning of NCD risk factors (mainly obesity) differs by level of urbanization [57, 58]. To our knowledge, one study [21] has investigated how urbanicity modifies educational gradients in NCD risk factors in Argentina. They found higher education to be more strongly associated with better risk factors profiles in more urban areas. Our results are in accordance with those reported by Fleischer et al. for ENFR_2005_ [21], but extend analyses to two additional surveys (ENFR_2009_ and ENFR_2013_). Despite efforts made to reduce educational inequalities in risk factors in Argentina (for example, through the implementation of national programs on adult education [59] and NCDs prevention [60, 61], our results suggest that there is more work to do to reduce what appear to be growing inequalities in NCD risks factors, especially in more urban areas.

An important limitation of our study is the use of self-reported outcomes, which can be affected by access to care especially in the case of diabetes and hypertension, and for lower educational groups [45, 62]. However, previous studies have demonstrated good agreement between objective and self-reported hypertension, diabetes [63] and smoking [64]. It is likely that our urbanicity indicator may have been a proxy for a variety of social and economic changes associated with urbanization in Argentina during the period of study. We did not attempt to disentangle urbanization itself from features with which urbanization may be correlated such as income, wealth, or inequalities. Future work should further explore the mechanisms behind the associations we observed with urbanization. Our study was based on comparable national health surveys in Argentina, with response rates above 70%. Furthermore, we estimated educational inequalities using the RII [26], ensuring the comparability of these estimates over time, all in a multilevel framework that account for clustering within province. Future work should examine whether some of the patterns that we report are further modified by age, as evidence suggests that the association of SEP with health weakens at older ages [65–67], although methodological issues including survivor bias, higher rates and their impacts on relative inequalities, and changes in the distribution of education may affect some of these results.

In summary, we found evidence that key NCD risk factors of obesity and diabetes are increasing over time in urban areas of Argentina. We also found large inequities by education especially in women with some inequities increasing over time. There was some evidence that inequities were larger in more urbanized provinces. These results highlight the need to focus on policies to reduce NCD risk factors among lower socioeconomic groups by modifying urban environments in Argentina.

## Data Availability

The anonymous data used in this study can be obtained from the National Institute of Statistics and Census of Argentina [‘Instituto Nacional de Estadísticas y Censos’ (INDEC); “https://www.indec.gob.ar/indec/web/Institucional-Indec-BasesDeDatos-2”].

## Abbreviations

BMI: Body mass index
NCDs: Non-communicable diseases
ENFR: Encuesta Nacional de Factores de Riesgo
RII: Relative index of inequality
SEP: Socioeconomic position
